# Deep learning‐based digitization of prostate brachytherapy needles in ultrasound images

**DOI:** 10.1002/mp.14508

**Published:** 2020-10-27

**Authors:** Christoffer Andersén, Tobias Rydén, Per Thunberg, Jakob H. Lagerlöf

**Affiliations:** ^1^ Department of Medical Physics Faculty of Medicine and Health Örebro University Örebro Sweden; ^2^ Department of Medical Physics and Biomedical Engineering Sahlgrenska University Hospital Gothenburg Sweden; ^3^ Department of Medical Physics Karlstad Central Hospital Karlstad Sweden

**Keywords:** brachytherapy, deep learning, high‐dose‐rate, image segmentation, needle digitization

## Abstract

**Purpose:**

To develop, and evaluate the performance of, a deep learning‐based three‐dimensional (3D) convolutional neural network (CNN) artificial intelligence (AI) algorithm aimed at finding needles in ultrasound images used in prostate brachytherapy.

**Methods:**

Transrectal ultrasound (TRUS) image volumes from 1102 treatments were used to create a clinical ground truth (CGT) including 24422 individual needles that had been manually digitized by medical physicists during brachytherapy procedures. A 3D CNN U‐net with 128 × 128 × 128 TRUS image volumes as input was trained using 17215 needle examples. Predictions of voxels constituting a needle were combined to yield a 3D linear function describing the localization of each needle in a TRUS volume. Manual and AI digitizations were compared in terms of the root‐mean‐square distance (RMSD) along each needle, expressed as median and interquartile range (IQR). The method was evaluated on a data set including 7207 needle examples. A subgroup of the evaluation data set (n = 188) was created, where the needles were digitized once more by a medical physicist (G1) trained in brachytherapy. The digitization procedure was timed.

**Results:**

The RMSD between the AI and CGT was 0.55 (IQR: 0.35–0.86) mm. In the smaller subset, the RMSD between AI and CGT was similar (0.52 [IQR: 0.33–0.79] mm) but significantly smaller (*P* < 0.001) than the difference of 0.75 (IQR: 0.49–1.20) mm between AI and G1. The difference between CGT and G1 was 0.80 (IQR: 0.48–1.18) mm, implying that the AI performed as well as the CGT in relation to G1. The mean time needed for human digitization was 10 min 11 sec, while the time needed for the AI was negligible.

**Conclusions:**

A 3D CNN can be trained to identify needles in TRUS images. The performance of the network was similar to that of a medical physicist trained in brachytherapy. Incorporating a CNN for needle identification can shorten brachytherapy treatment procedures substantially.

## 1 INTRODUCTION AND PURPOSE

**Fig 1:**
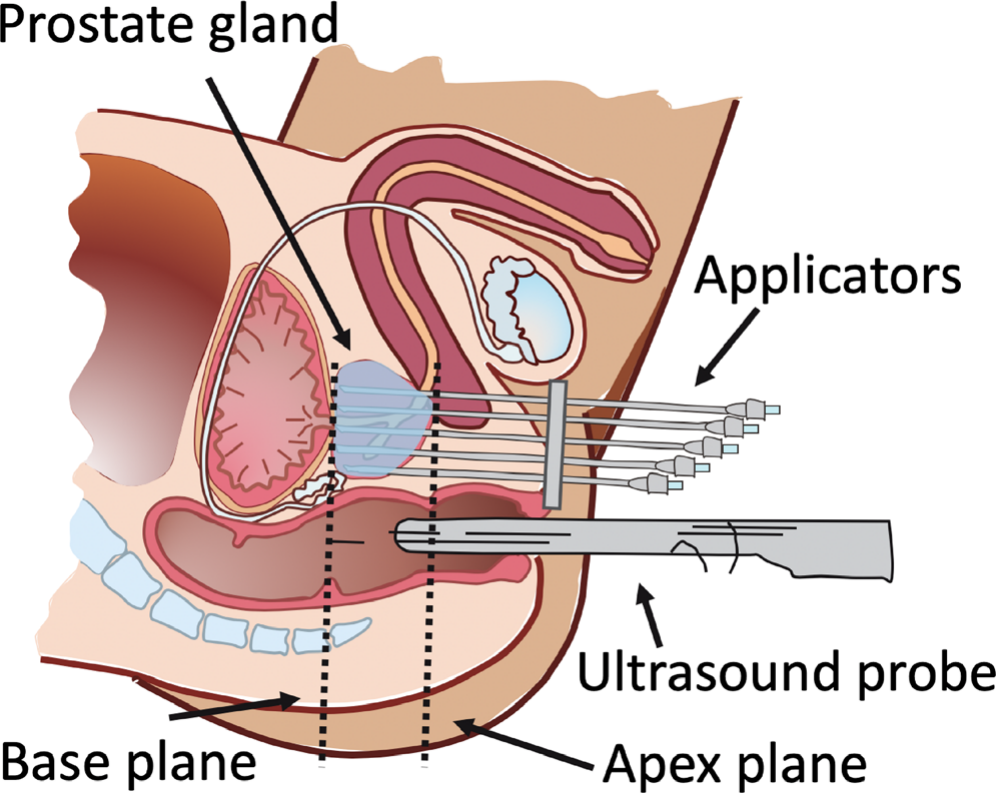
Illustration of the prostate in the sagittal plane with needles in a brachytherapy treatment setting for prostate cancer. The transrectal ultrasound probe is used to visualize the prostate gland and the needles in place. [Color figure can be viewed at wileyonlinelibrary.com]

Radiation treatment options for stage T1b–T3b prostate cancer include external beam therapy, brachytherapy, or a combination of these. Brachytherapy is a procedure where the radiation source is placed directly into or near the volume to be treated. For prostate cancer, the radiation source is placed inside the prostate. Hollow needles are surgically inserted transperineally into the prostate under direct guidance of transrectal ultrasound (TRUS);^1^ a minimum of 13 needles is recommended.^2^ In these needles, an afterloaded radiation source is positioned at certain locations for different periods of time to create a dose distribution. An illustration of the setup is shown in Fig. 1.

In high‐dose‐rate brachytherapy (HDRBT), the dose is delivered continuously over a short period of time lasting just a few minutes. The patient is under anesthesia during the whole treatment, which can last almost 4 h.^3^

The afterloaded radiation source is Iridium‐192, which has been used in brachytherapy since the 1980s^4^ and is still in common use for this purpose. One of its advantages is a steep dose gradient.^5^ Before treatment planning, it is essential to accurately digitize the implanted needles^6^ in order to avoid over‐dosage of the organs‐at‐risk and under‐dosage of the prostate volume. Needle digitization is a critical step in HDRBT for prostate cancer with respect to the outcome of the dose plan.

The digitization is performed by a medical physicist or a dosimetrist who acquires this expertise over time by practising the task. This means not only that the digitizing process is subjective and may vary between operators, but also that depending on their experience the operator may spend a lot of time on this important task. There is a strong need to automate the digitization process, potentially improving both speed and reproducibility.

Deep learning, which is a branch of artificial intelligence (AI), has progressed rapidly in various healthcare areas. For example, convolutional neural networks (CNNs), which are a type of deep learning algorithm, have had a major impact on image processing.^7^, ^8^ Long et al.^9^ made the first breakthrough in this area, with an application of fully convolutional networks that achieved state‐of‐the‐art segmentation results via end‐to‐end training. A neural network uses a set of algorithms designed to recognize numerical patterns in a very similar way to how the human brain works. The network practises on numerous examples and gets progressively better.^10^

Deep learning for segmentation in ultrasound images has previously been studied by others.^11^, ^12^, ^13^, ^14^, ^15^, ^16^, ^17^ Two recent studies using, in the field of prostate brachytherapy, deep learning has been utilized for needle digitization in prostate brachytherapy^18^, ^19^ trained the algorithm using patches instead of the whole image volume, and employed a weighted loss function between cross entropy and total variation for optimization. However, other metrics exist describing intersection over union, such as the Dice similarity coefficient. The application of the Dice coefficient as a metric in the optimization process and the use of the whole TRUS image volume as input constitute a new alternative in AI digitization of needles in prostate brachytherapy.

From a clinical perspective, it is of interest to elucidate the performance of a CNN in relation to the manual digitization of needles performed by different operators. This is commonly studied in terms of interobserver variability, and is also applicable when evaluating the results provided by the AI. A likely advantage of employing AI in brachytherapy in clinical practice is the reduced treatment time due to the almost instant digitization of the needles provided by the AI compared to the manual digitization carried out by the human operator.

The aims of this study were to develop a three‐dimensional (3D) CNN algorithm for finding needles in TRUS image volumes and to evaluate its performance in relation to manual digitization.

## 2 MATERIALS AND METHODS

The Regional Research Ethics Board approved this retrospective study and waived informed consent.

### 2.1 Data collection

**Fig 2:**
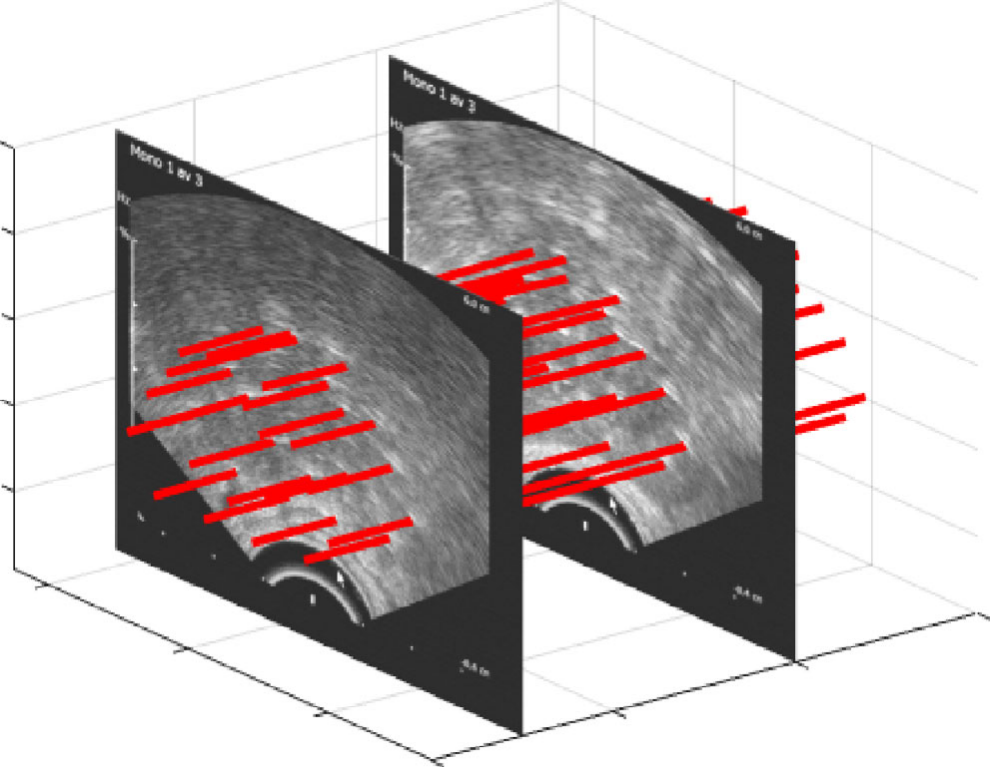
Ultrasound images encompassing the complete prostate gland with margins, acquired after a sweep of the transrectal ultrasound, yielding a three‐dimensional volume including 20 needles (highlighted with red lines). [Color figure can be viewed at wileyonlinelibrary.com]

The data were drawn from 1102 brachytherapy treatments of prostate cancer performed at Örebro University Hospital between 2010 and 2019. Each treatment included a set of 2D axial TRUS images encompassing the complete prostate gland, with margins, and the inserted needles. The set of 2D TRUS images created a 3D volume in which the needles were located (Fig. 2).

The interslice distance of the TRUS images was 1 mm, and there was no gap between adjacent slices. Each image volume consisted of 512 × 512 images with a pixel size of $0.129\;\times \;0.129\;{\text{mm}}^{2}$.

Digitization of needles seen in the 3D image volume was performed by a medical physicist during treatment using the software provided by the vendor (Oncentra Prostate versions 4.2.2–4.2.21, Elekta AB, Stockholm, Sweden). Two types of ultrasound equipment were used: Pro Focus (B‐K Medical) with type 8658 probes and Prosius (Elekta) with BiopSee7 5/70/128 probes. Steel needles provided by the vendor (Elekta AB) were used as applicators. When inserted into the prostate, the needles were overshot by 10 mm to avoid dwell positions near the needle tip. Two semi‐orthogonal radiograph images were used to measure the depth of the needle length.

All needles that had been manually digitized by a medical physicist composed a data set considered to be the clinical ground truth (CGT). When digitizing, the medical physicist assumed that the needles were straight at all times except in the very rare cases where they were considered to be slightly bent.

When performing a brachytherapy treatment for prostate cancer, the prostate and organs at risk are segmented (in this case using Oncentra Prostate), and the decision is made on which images to include in the treatment planning. In this study we decided to include images ranging from 20 mm superior of the base of the prostate to 10 mm inferior of the apex of the prostate for all treatments. The base and apex planes of the prostate are indicated in Fig. 1.

The prostate gland has the shape of a walnut and dimensions that range from approximately 20–60 mm,^20^ and so the expected depth of the 3D TRUS image volume should range from approximately 50–90 mm, including the additional margins relative to the base and apex planes. For this study, it was decided to create a standard cubic 3D image volume size of 90 mm that encompassed all treatment volumes. Acquired 3D TRUS image volumes smaller than 90 mm were zero padded to form a $90\;\times \;90\;\times \;90\;{\text{mm}}^{3}$ cubical volume. Thus, all TRUS volumes had the same sizes and were resampled to yield 128 × 128 × 128 voxels. The data were randomly distributed between training (65%), validation (17.5%), and testing (17.5%).

### 2.2 Network structure

**Fig 3:**
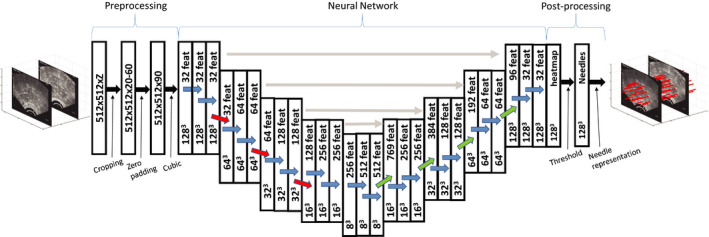
Graphical illustration of the applied three‐dimensional U‐net with a 128 × 128 × 128 TRUS image volume as input and the needle localizations in the volume as output. Convolutions (blue arrows), maxpooling (red arrows), and transposed convolutions (green arrows) were performed using 3 × 3 × 3 kernels. The gray horizontal arrows illustrate the concatenation of data of feature maps (feat) from the contraction path to the expansion path. [Color figure can be viewed at wileyonlinelibrary.com]

A U‐net structure^21^ of the CNN was used for segmenting the needles in the TRUS images. This structure is widely used for segmentation in medical imaging.^21^, ^22^, ^23^, ^24^ The 3D U‐net structured CNN used in the present study is illustrated in Fig. 3.

The input used in the 3D U‐net was the 128 × 128 × 128 3D ultrasound image volumes. The 3D volumes were convolved with a 3 × 3 × 3 filter kernel using wide convolution, after which a rectified linear unit was applied. This process was repeated once again, and then the 3D images were downsampled via maxpooling, using a 2 × 2 × 2 filter. The process, including two filters, two rectified linear units and maxpooling, was carried out four times, resulting in matrix dimensions of 8 × 8 × 8. In this way, the essential information in the image was extracted before the 3D U‐net started the upsampling back to 128 × 128 × 128. The upscaling process used a transposed convolutional layer. A drop out ratio of 0.2 was used to avoid overfitting. During upsampling, the output was concatenated with the corresponding level matrix during the decoding phase.

### 2.3 Training

During the training process, 713 treatments were used with 15–25 needles per treatment. The hyperparameters remained constant during the training. Initial weights for the 3D U‐net network were chosen with the Glorot uniform initializer.^25^ The network was optimized by maximizing the Dice similarity coefficient, DSC (Eq. 1), between the predicted needles and the CGT localization performed by a medical physicist during treatment.

(1) $$DSC=2\frac{\left|X\cap Y\right|}{\left|X|+|Y\right|}$$

In Eq. (1), X and Y are given sets with cardinalities |*X*| and |*Y*|, for the manually and algorithmically segmented needles. DSC was calculated for all pixels. The deep 3D U‐net architecture was implemented in CNTK 2.6 using an NVIDIA GTX 2080Ti GPU. The model was trained for 50 epochs with a batch size of 1. Each epoch took 15 min with this setup. The Adam optimizer was used with a learning rate decreasing linearly from 1.2e‐5 to 8e‐6. No data augmentation was deemed necessary due to the large data set.

### 2.4 Merging AI predictions into a needle representation

The output from the AI consisted of predictions, ranging from zero to one, of whether or not each voxel corresponded to a needle. These predictions were used as weights to calculate one coordinate of a needle position in each slice. The probability map was thresholded at 0.1. The center coordinates (x and y) were calculated for each needle and for each slice, and a standard least‐square fit (LS) of all included coordinates then yielded a 3D linear equation for each needle through the TRUS volume. This needle equation constituted the AI description of the needle, and could be compared to the manually digitized needle, which was also described as a 3D linear function in the dose planning system. The equations of the manually digitized needles were extracted from the DICOM headers in the ultrasound images.

### 2.5 Evaluation of the algorithm

A set of 389 different treatments was used to evaluate the neural network. The total number of needles digitized was 7207. These data were never used for training. The cases (treatments) were digitized by the algorithm and compared to the CGT digitized by medical physicists via examination of the root‐mean‐square deviation (RMSD) and statistical analysis.

### 2.6 Geometric evaluation

To estimate how much the AI digitization deviated from the CGT, each needle’s RMSD was calculated using Eq. (2), where *δ* is the distance from the AI digitization in the slice *i* to the CGT digitization in the same slice. The first slice (*i* = 1) started 8 mm inside the prostate from the apex plane, and *N* was 27, meaning that the last slice was 35 mm from the apex and

(2) $$\text{RMSD}=\frac{\sqrt{\sum_{i=1}^{N}\delta_{i}^{2}}}{N}$$

the RMSD was calculated for a total distance of 27 mm along the needles. This distance was mainly located inside the prostate where the treatment took place, and was consequently considered to be the region where the digitization was of most importance.

The angular deviation (AD) between two vector representations of a needle (**LS** and ${\mathrm{LS}}_{\mathrm{ref}}$) was calculated according to Eq. (3)

(3) $$\text{AD}=\text{arccos}\left(\frac{\mathrm{LS}\cdot {\mathrm{LS}}_{\mathrm{ref}}}{\left\|\mathrm{LS}\|\right\|{\mathrm{LS}}_{\mathrm{ref}}\|}\right).$$

### 2.7 Interobserver variability

To test the interobserver variability between human operators and the performance of the AI against a new operator, an additional set of CGT data consisting of nine treatments (188 needles) was redigitized by another medical physicist. This new data set, which was denoted G1, was a subset of the CGT data set.

### 2.8 Duration of manual digitization of needles

The duration of the redigitization of the small data set (G1) was measured, and the mean value was calculated to estimate the time consumption per treatment for manual digitization. This was compared to the time spent to digitize the data with AI.

### 2.9 Statistical analysis

Visual inspection showed the RMSD distributions to be log‐normal. The logarithms of the distributions were calculated and Student’s t test was applied to ${\text{RMSD}}_{\mathrm{AI},\mathrm{CGT},\;n\;=\;188}$ and ${\text{RMSD}}_{\mathrm{AI},\mathrm{CGT},\;n\;=\;7207}$ to determine whether a sample size of 188 was large enough to represent the whole data set. Furthermore, a t test was applied to ${\text{RMSD}}_{\mathrm{AI},\mathrm{CGT},\;n\;=\;7207}$ and each of ${\text{RMSD}}_{\mathrm{AI},G1,\;n\;=\;188}$ and ${\text{RMSD}}_{\mathrm{CGT},G1,\;n\;=\;188}$, respectively, to estimate whether or not the interobserver variability between AI and CGT was significantly different from that between G1 and CGT. A *P* < 0.05 was considered to be significant. Data, when not log‐normal, are presented using the median and interquartile range (IQR) in the form of median (IQR).

## 3 RESULTS

**Fig 4:**
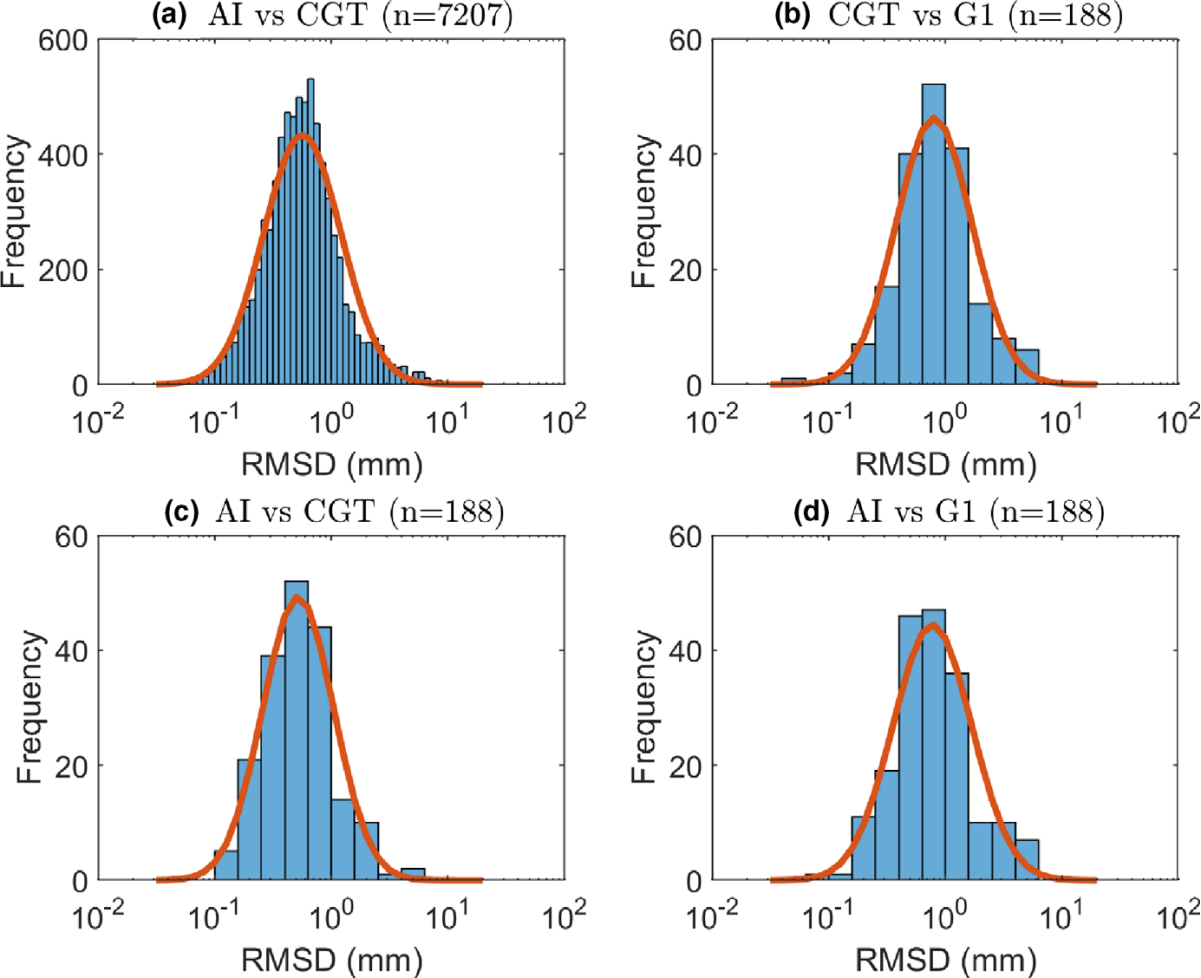
Logarithm histograms for (a) ${\text{RMSD}}_{\mathrm{AI},\mathrm{CGT},\;n\;=\;7207}$, (b) ${\text{RMSD}}_{\mathrm{CGT},G1,n\;=\;188}$, (c) ${\text{RMSD}}_{\mathrm{AI},\mathrm{CGT},\;n\;=\;188}$, and (d) ${\text{RMSD}}_{\mathrm{AI},G1,n\;=\;188}$. A normal distribution adapted to the data is shown as a red curve for each histogram. [Color figure can be viewed at wileyonlinelibrary.com]

The DSC plateaued at 0.5 after 10 epochs for the validation set. The detection of the needles was 100%. Figure 4 shows differences expressed as RMSD for various comparisons between AI and manual digitizations of the needles in TRUS image volumes. The median and IQR of the RMSD data shown as lognormal histograms in Fig. 4 were 0.55 (0.35–0.86) mm for ${\text{RMSD}}_{\mathrm{AI},\mathrm{CGT},\;n\;=\;7207}$, 0.80 (0.48–1.18) mm for ${\text{RMSD}}_{\mathrm{CGT},G1,\;n\;=\;188}$, 0.52 (0.33–0.80) mm for ${\text{RMSD}}_{\mathrm{AI},\mathrm{CGT},\;n\;=\;188}$ and 0.75 (0.49–1.20) mm for ${\text{RMSD}}_{\mathrm{AI},G1,\;n\;=\;188}$.

There was no statistically significant difference between ${\text{RMSD}}_{\mathrm{AI},\mathrm{CGT},\;n\;=\;7207}$ [Fig. 4(a)] and ${\text{RMSD}}_{\mathrm{AI},\mathrm{CGT},\;n\;=\;188}$ [Fig. 4(c)]. This implies that the AI performs equally well in repeated digitization tasks, a kind of intra‐AI variability test. Conversely, there was a significant difference between ${\text{RMSD}}_{\mathrm{AI},\mathrm{CGT},\;n\;=\;188}$ and ${\text{RMSD}}_{\mathrm{AI},G1,n\;=\;188}$ [Fig. 4(d)], indicating that the AI had learned to digitize like the CGT and hence perform differently from G1. This was confirmed by similar difference between ${\text{RMSD}}_{\mathrm{AI},\mathrm{CGT},\;n\;=\;188}$ and ${\text{RMSD}}_{\mathrm{CGT},G1,n\;=\;188}$ [Fig. 4(b)].

**Fig 5:**
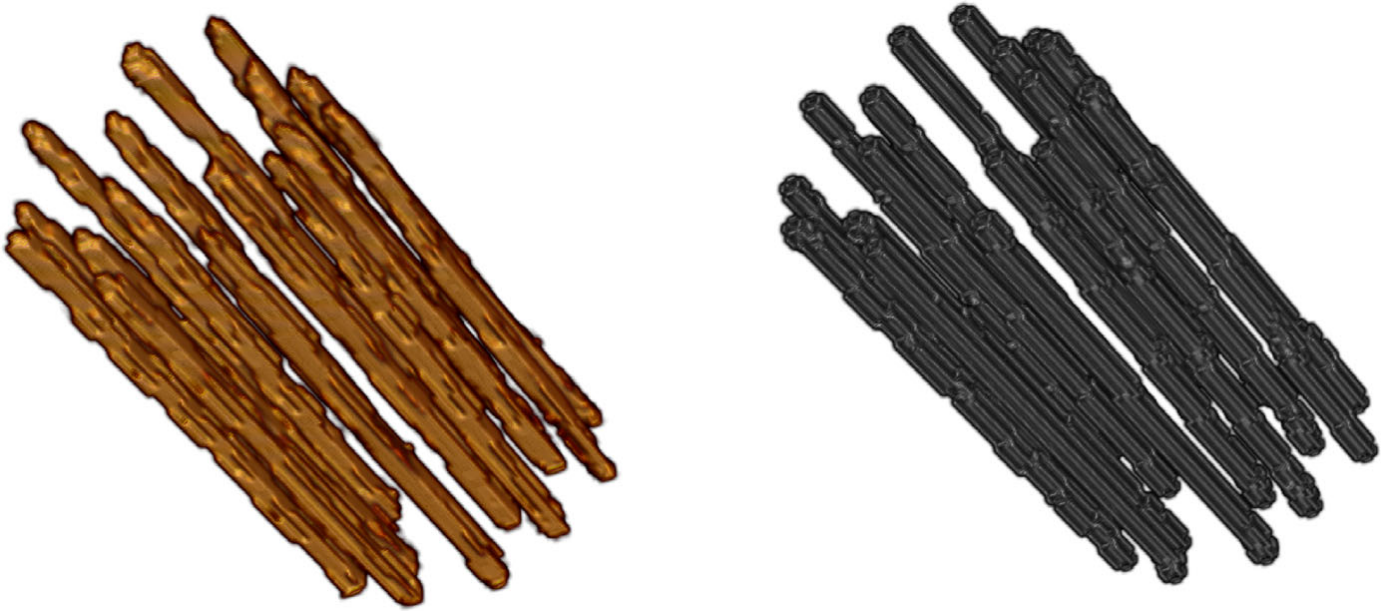
Visual comparison between artificial intelligence (AI) digitization (left panel) and the clinical ground truth (right panel). The AI digitization is represented by expectation values with an isosurface of 0.1 [Color figure can be viewed at wileyonlinelibrary.com]

The results of this analysis are summarized in Table I. The median and IQR of the AD were $0.88^{\circ }$ ($0.52^{\circ }$  $‐1.41^{\circ }$) for ${\text{AD}}_{\mathrm{AI},\mathrm{CGT},\;n\;=\;188}$, $0.93^{\circ }$ ($0.55^{\circ }$   $‐1.48^{\circ }$) for ${\text{AD}}_{\mathrm{AI},G1,\;n\;=\;188}$, and $1.00^{\circ }$ ($0.62^{\circ }$   $‐1.57^{\circ }$) for ${\text{AD}}_{\mathrm{CGT},G1,\;n\;=\;188}$. A visual comparison between the AI digitization and clinical ground truth is shown in Fig. 5. The AI digitizations seen in the figure are expectation values with an isosurface of 0.1.

### 3.1 Duration of manual digitization of needles

The average time spent on manual digitization was 10 min 11 s ± 2 min 38 s per treatment. The normal use of needles at the clinic is 20–25 needles per treatment. The time spent on the AI digitization was negligible; <2 s for our implementation and equipment.

**Table I:** The performance of the artificial intelligence (AI) was evaluated in terms of root‐mean‐square deviation (RMSD) by comparing the AI to the clinical ground truth (CGT) for both the test data set (n = 7207) and a smaller subgroup (n = 188) of the test data for which manual digitization was repeated by another medical physicist (G1)

	**RMSD [mm]**	**Significance**
	median (IQR)	
**AI vs CGT (n = 7207)**	0.55 (0.35–0.86)	
**AI vs CGT (n = 188)**	0.52 (0.33–0.79)	*P* = 0.15
**AI vs G1 (n = 188)**	0.75 (0.49–1.20)	*P* < 0.0001
**CGT vs G1 (n = 188)**	0.80 (0.48–1.18)	*P* < 0.0001

The test data set was used as reference for statistical comparisons except for clinical ground truth (CGT) vs G1.

## 4 DISCUSSION

Deep learning in brachytherapy is a rapidly growing area of research. Previous studies include work on digitizing gynaecological applicators,^6^, ^26^, ^27^ prostate seeds,^28^, ^29^ and segmentation of the prostate boundaries.^30^, ^31^, ^32^ Recent publications by Zhang et al.^18^, Wang et al.^33^ and Dise et al.^26^ have used deep learning for identifying needles in 3D TRUS images.

The performance of a CNN relies on the extent of the training data, and can be evaluated using different measures. This study used a large set of 1102 treatments and can be compared to previous studies that used far less data^6^, ^18^, ^26^, ^28^, ^29^ (13, 10, 68, 13, and 23 patients, respectively). However, one recent study^33^ used 823 patients. The AI localizations of needles were compared to both the labeled data and localizations by an additional medical physicist, thus enabling an intervariability comparison between a trained AI and a human. The performance of the AI was, as expected, similar to the CGT, but the intervariability differed significantly compared to G1. Since the difference between CGT and G1 was similar to the difference between AI and G1, it seems that an AI can be trained to act as an experienced medical physicist and accordingly perform similarly to other medical physicists in terms of intervariability. The results in our study are similar to those from other studies using CNNs with 3D U‐net architecture for needle segmentation in prostate brachytherapy in ultrasound images.^18^, ^26^, ^33^, ^34^ Wang et al.^33^ used a large training set and reported an RMSD of 0.74 mm, which is slightly higher than the 0.55 mm found in the present study. Zhang et al.^18^, ^34^ and Dise et al.^26^ both reported lower RMSD (0.29 and 0.40 mm) than achieved in this study, but neither were able to reach the 100% sensitivity which is the case for the present study. One difference between the algorithm in this article and those found in the literature is that the present algorithm is optimized with DSC.

The present study also evaluated the angular deviation between the manual and algorithm segmented needles. The median angular deviation between the clinical ground truth and the algorithm was within $1^{\circ }$, as was the angular deviation for the intervariability.

Because the straight needles are composed of the weighted sum, shadowing on the needles did not present a problem. Both the medical physicist in the clinic and the algorithm assumed that the needles were straight. This could be one reason for the angular deviation of $0.88^{\circ }$, which we consider to be low. A useful comparison can be made with the results of Wang et al.^33^ who also had a perfect sensitivity and included a large training set, but had a RMSD somewhat higher as opposed to the result in the present study. The difference between the two studies is the optimization and the assumption of straight or nonstraight needles, and so the results in the present study indicate that assuming straight needles is sufficient.

The present study also evaluated the time spent on digitizing the needles. For a human operator, the mean time spent was 10 min 11 s per patient. Nicolae et al. presented a mean time of 7.50 min per patient with 12–16 needles,^35^ which would mean 10 min 42 s per patient if the patient had 20 needles. This is well in line with the time for manual digitization measured in this study.

To our knowledge, no previous study has evaluated the performance of an AI in relation to manual interobserver variability regarding the digitization of needles in ultrasound images for prostate brachytherapy. The present study has shown that the implemented AI performed equivalently to the interobserver difference between two human operators. This way of evaluating an AI can preferably be used as a benchmark in future studies including development and implementation of AI methods in imaging‐based brachytherapy.

The present study uses a network architecture called 3D U‐net, which has been used in previous studies involving deep learning in brachytherapy.^6^, ^18^, ^27^, ^30^, ^36^ The U‐net structure has been used for segmentation of both needles and organs such as the prostate.^18^, ^30^ A similar structure called V‐net has also been proposed, and this too has been used for segmentation of the prostate.^32^

This study does have some limitations. The deep neural network did not find the tips of the needles. The algorithm was never trained to do this, which could be considered a limitation. Usually, however, the tip is not digitized using a TRUS, but instead an x‐ray image is used to measure the distance from the base plane to the tip and this is manually entered into the system. The CGT was used when calculating the RMSD, but it must be remembered that the CGT is not the same as the absolute true positions of the needles. Since an imaging system like TRUS has its limitations when depicting an object in terms of resolution and image quality, it is difficult to say whether the AI is better than a traditional operator at digitizing needles. Nevertheless, we can say that AI shows a smaller RMSD than G1, which implies that AI behaves more like experienced medical physicists than G1 does. We have not been able to find any comparable values in the literature for RMSD of AI vs a human operator for this application. To investigate the precision of the ultrasound, the TRUS images could be fused with images acquired using computed tomography. From experience, we know that the needle in the TRUS tends to bend at the tip of the needle. The RMSD was not calculated along the whole needle, but only using the slices that encompassed the prostate. The ultrasound images used in this study were acquired using two different ultrasound systems. It is possible that the AI would perform less well if another ultrasound system was used in the TRUS acquisition, as the algorithm might have only learnt how to digitize needles in the present images and would therefore fail if used with another ultrasound model.

Future work aims to evaluate the difference in dosimetry between manually digitized and AI digitized needles.

## 5 CONCLUSIONS

We have developed a convolutional neural network via deep learning to digitize needles in prostate HDR brachytherapy contexts. The network demonstrated a precision that was higher than the interobserver variability.

## CONFLICT OF INTEREST

The authors have no conflict to disclose.
